# The Efficacy and Hemorheological Indexes of Ginseng and Its Active Components for Patients with Non-Small Cell Lung Cancer: A Systematic Review and Meta-Analysis

**DOI:** 10.1155/2023/3144086

**Published:** 2023-02-17

**Authors:** Yawen Xia, Hongkuan Han, Renjun Gu, Ruizhi Tao, Keqin Lu, Zhiguang Sun, Sanbing Shen, Aiyun Wang, Yin Lu

**Affiliations:** ^1^Jiangsu Key Laboratory for Pharmacology and Safety Evaluation of Chinese Materia Medica, School of Pharmacy, Nanjing University of Chinese Medicine, Nanjing 210023, China; ^2^Jiangsu Provincial Second Chinese Medicine Hospital, The Second Affiliated Hospital of Nanjing University of Chinese Medicine, Nanjing 210017, China; ^3^Regenerative Medicine Institute, School of Medicine, University of Galway, Biomedical Science Building BMS-1021, Dangan, Upper Newcastle, Galway, Ireland; ^4^Jiangsu Collaborative Innovation Center of Traditional Chinese Medicine Prevention and Treatment of Tumor, Nanjing University of Chinese Medicine, Nanjing 210023, China

## Abstract

**Background:**

Non-small cell lung cancer (NSCLC) is still a slightly less orphan disease after immunotherapy, and routine treatment has low efficiency and adverse events. Ginseng is commonly used in the treatment of NSCLC. The purpose of this study is to assess the efficacy and hemorheological indexes of ginseng and its active components in patients with non-small cell lung cancer.

**Methods:**

A comprehensive literature search was performed in PubMed, the Cochrane Library, Medline (Ovid), the Web of Science, Embase, CKNI, Wan Fang, VIP, and SinoMed up to July 2021. Only randomized controlled trials evaluating ginseng in combination with chemotherapy versus chemotherapy alone in NSCLC patients were included. Primary outcomes included patients' condition after using ginseng or its active components. Secondary outcomes included changes in immune cells, cytokines, and secretions in serum. Data were extracted by two independent individuals, and the Cochrane Risk of Bias tool version 2.0 was applied for the included studies. Systematic review and meta-analysis were performed by RevMan 5.3 software.

**Results:**

The results included 1480 cases in 17 studies. The results of the integration of clinical outcomes showed that the treatment of ginseng (or combination of ginseng with chemotherapy) can improve the quality of life for patients with NSCLC. Analysis of immune cell subtypes revealed that ginseng and its active ingredients can upregulate the percentages of antitumor immunocyte subtypes and downregulate the accounts of immunosuppressive cells. In addition, a reduction of the inflammatory level and an increase of antitumor indicators in serum were reported. Meta-analysis showed that Karnofsky score: WMD = 16, 95% CI (9.52, 22.47); quality-of-life score: WMD = 8.55, 95%CI (6.08, 11.03); lesion diameter: WMD = −0.45, 95% CI (−0.75, −0.15); weight: WMD = 4.49, 95% CI (1.18, 7.80); CD3^+^: WMD = 8.46, 95% CI (5.71, 11.20); CD4^+^: WMD = 8.45, 95% CI (6.32, 10.57)+; CD8^+^: WMD = −3.76, 95% CI (−6.34, −1.18); CD4^+^/CD8^+^: WMD = 0.32, 95% CI (0.10, 0.53); MDSC: WMD = −2.88, 95% CI (−4.59, −1.17); NK: WMD = 3.67, 95% CI (2.63, 4.71); Treg: WMD = −1.42, 95% CI (−2.33, −0.51); CEA: WMD = −4.01, 95% CI (−4.12, −3.90); NSE: WMD = −4.00, 95% CI (−4.14, −3.86); IL-2: WMD = 9.45, 95% CI (8.08, 10.82); IL-4: WMD = −9.61, 95% CI (−11.16, −8.06); IL-5: WMD = −11.95, 95% CI (−13.51, −10.39); IL-6: WMD = −7.65, 95% CI (−8.70, −6.60); IL-2/IL-5: WMD = 0.51, 95% CI (0.47, 0.55); IFN-*γ*: WMD = 15.19, 95% CI (3.16, 27.23); IFN-*γ*/IL-4: WMD = 0.91, 95% CI (0.85, 0.97); VEGF: WMD = −59.29, 95% CI (−72.99, −45.58); TGF-*α*: WMD = −10.09, 95% CI (−12.24, −7.94); TGF-*β*: WMD = −135.62, 95% CI (−147.00, −124.24); TGF-*β*1: WMD = −4.22, 95% CI (−5.04, −3.41); arginase: WMD = −1.81, 95% CI (−3.57, −0.05); IgG: WMD = 1.62, 95% CI (0.18, 3.06); IgM: WMD = −0.45, 95% CI (−0.59, −0.31). All results are statistically significant. No adverse events were reported in the included articles.

**Conclusion:**

It is a reasonable choice to use ginseng and its active components as adjuvant therapy for NSCLC. Ginseng is helpful for NSCLC patients' conditions, immune cells, cytokines, and secretions in the serum.

## 1. Introduction

According to the latest data released by the World Health Organization's International Agency for Research on Cancer (IRAC) in 2020, lung cancer is one of the most common cancers with a high mortality rate. It can be divided into non-small cell lung cancer (NSCLC) and small cell lung cancer [1]. The former accounts for about 85% [2, 3]. NSCLC is still a slightly less orphan disease after immunotherapy [4]. Platinum-based chemotherapy after surgery is still the standard treatment for patients with resectable, nonmetastatic, non-small cell lung cancer [5]. In recent years, the advent of targeted drugs and immunotherapy has given new hope to NSCLC patients [6–8]. However, low efficiency and high costs of treatment remain huge problems.

Ginseng is a traditional Chinese herb and is the dried root and rhizome of Panax ginseng. It has been used for more than two thousand years as a traditional tonic medicine. Ginseng contains a lot of pharmacologically active ingredients, such as ginsenosides Rb1, Rb2, Rg3, ginseng polysaccharides, etc. [9], which are often used in neurasthenia [10], psychosis, cardiovascular system diseases [11], and diabetes [12]. It also widespread administrated in NSCLC treatment plans [13]. Ginseng shows the highest usage frequency (about 32.5%) among 110 commonly used traditional herbs for lung cancer [14].

It was reported that ginseng and its ingredients have tumor-killing and metastasis-preventing potentials. For example, ginsenoside Rg3 can induce DNA damage by activating the VRK1/P53BP1 pathway to reduce the occurrence of NSCLC [15], and the total extract of ginseng can activate the endoplasmic reticulum stress through the ATF4-CHOP-AKT1-mTOR axis to induce autophagic cell death [16]. In addition, ginseng and its active components are often used to enhance chemotherapy sensitivity and alleviate adverse symptoms [17, 18]. Related mechanisms may be involved in triggering apoptosis in human lung adenocarcinoma cells, promoting macrophages' transformation from type M2 to type M1, and keeping balance between Th1/Th2 T-helper cells [18–21].

At present, some clinical trials explore the effects of ginseng. However, clinical trials found that a ginseng-related medicine with navelbine and cisplatin chemotherapy had no significant changes on patients' 1-year survival rates [22]. The function of ginseng in non-small cell lung cancer is still uncertain. Therefore, we will conduct this systematic review and meta-analysis to assess the efficacy and hemorheological indexes of ginseng and its active components on patients with non-small cell lung cancer.

## 2. Information and Methods

### 2.1. Study Protocol

This systematic review and meta-analysis followed the Preferred Reporting Items for Systematic Reviews and Meta-Analyses (PRISMA) of 2015 guideline [23].

### 2.2. Search Strategy

Electronic literature searches were performed in the databases of PubMed, the Cochrane library, the Medline (Ovid), Web of Science, Embase, CKNI, Wan Fang, VIP, and SinoMed up to July 2021. Search strategy of Medline (Ovid) is as follows:

  #1. exp panax/.
  #2. ginseng.tw.
  #3. panax.tw.
  #4. or/1–3.
  #5. exp small cell lung cancer/.
  #6. oat cell.tw.
  #7. SCLC.tw.
  #8. or/5–7.
  #9.4 and 8.

### 2.3. Inclusion Criteria

Inclusion criteria were as follows: (a) randomized controlled trials (RCTs); (b) inclusion of people diagnosed with non-small cell lung cancer [24]; (c) interventions using ginseng or its active components as the main treatment. The combination therapy of ginseng or its active components and other interventions compared with the same other interventions alone was also included; and (d) included studies do not have any language limits.

### 2.4. Exclusion Criteria

Exclusion criteria were as follows: (a) non-clinical studies (experimental and basic studies); (b) observational or retrospective studies; and (c) lack of sufficient information on baseline or primary or secondary outcome data.

### 2.5. Primary Outcome

Changes in patients' conditions after using ginseng or its active components, such as Karnofsky score, quality-of-life score, lesion diameter, and weight.

### 2.6. Secondary Outcomes

1. Any changes in immune cells, such as CD3^+^, CD4^+^, CD8^+^, CD4^+^/CD8^+^, MDSC, NK, or Treg.
2. Any changes in cytokines and secretions in the serum, such as CEA, NSE, IL-2, IL-4, IL-5, IL-6, IL-2/IL-5, IFN-*γ*, IFN-*γ*/IL-4, VEGF, TGF-*α*, TGF-*β*, TGF-*β*1, arginase, IgG, and IgM.

### 2.7. Patient and Public Involvement

Neither patients nor the public were involved in the design of this study. This systematic review and meta-analysis did not recruit any patients.

### 2.8. Data Collection

Data were extracted by two independent reviewers (YX; HH). We consulted a third review author (RG) when we had any disagreements.

### 2.9. Bias Risk Assessment

According to the risk of bias assessment tool from the Cochrane Handbook [25] for Systematic Reviews of Interventions, Version 6.0 (updated July 2019) [26], two authors independently assessed the risk of bias of the included study, and any conflicts were resolved through consensus. Bias risk assessment was evaluated using the following seven items: random sequence generation, assignment concealment, blinding of participants and personnel, blinding of outcome assessment, incomplete outcome data, selective reporting, and other biases. These items are described as green, yellow, and red colors and “+,” “−,” “?.” The symbols indicate “low,” “high,” and “unclear” risk of bias.

### 2.10. Statistical Analysis

We followed the methods of Gu et al. [27]. The statistical analyses were performed by using Review Manager software (RevMan version 5.3, Cochrane Collaboration, Oxford, UK). Weighted mean difference (WMD) and 95% CI were used as the effect quantity to merge the continuous variables included in the study. *I*^2^ statistic will be used to test for heterogeneity between trial results. The random effect model was used when *I*^2^>50% according to the clinical heterogeneity. The statistical calculation process was completed by RevMan5.3 software [28, 29].

## 3. Results

### 3.1. Literature Search

**Figure 1:**
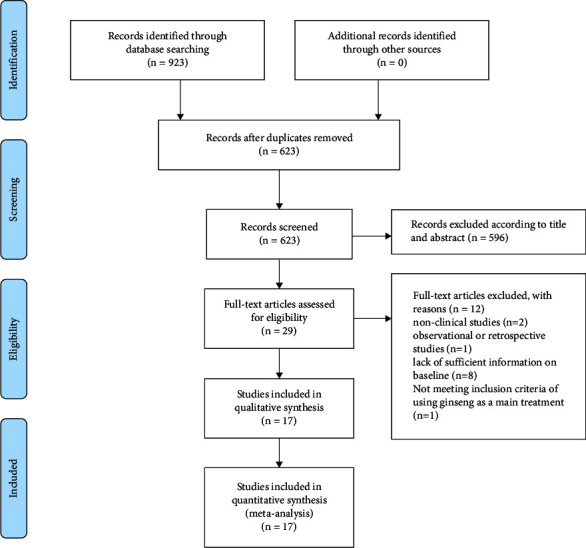
Flowchart of study selection.

Initial searches generated 923 related studies. According to the inclusion criteria and exclusion criteria, 29 studies were included for full-text consideration. Finally, 17 studies are included for meta-analysis. All studies are non-English studies. (See Figure 1).

### 3.2. Characteristics of the Study

17 articles were included in the study (see Table 1).

### 3.3. Risk of Bias

**Figure 2:**
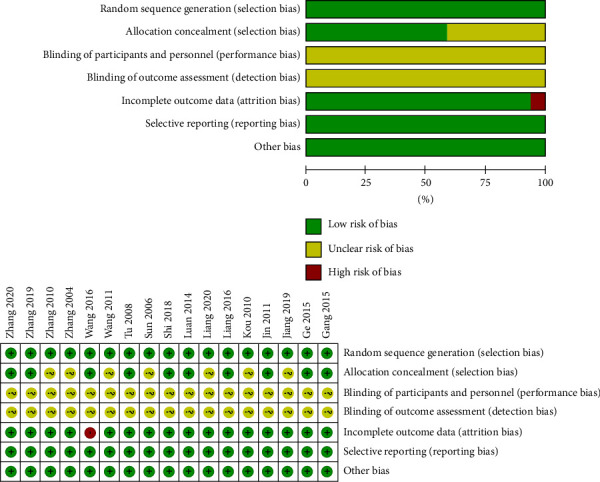
Quality assessment of the included studies.

The results of the risk of bias assessment of the 17 studies were summarized in Figure 2. All of them did not describe performances bias and detection bias.

### 3.4. Changes of Patients' Condition

#### 3.4.1. Karnofsky Score

**Figure 3:**
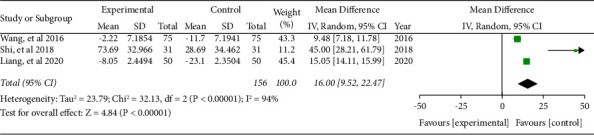
Forest plot of Karnofsky score.

Three literature included the Karnofsky Score. The combined effect was WMD = 16, 95% CI (9.52, 22.47), *P* < 0.05. The data were statistically significant (see Figure 3).

#### 3.4.2. Quality-of-Life Score

**Figure 4:**
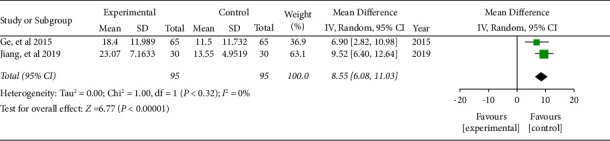
Forest plot of quality-of-life score.

Two literature included the quality-of-life score. The combined effect was WMD = 8.55, 95% CI (6.08, 11.03), *P* < 0.05. The data were statistically significant (see Figure 4).

#### 3.4.3. Lesion Diameter

**Figure 5:**
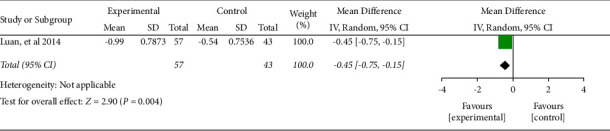
Forest plot of lesion diameter.

One literature included the lesion diameter. The combined effect was WMD = −0.45, 95% CI (−0.75, −0.15), *P* < 0.05. The data were statistically significant (see Figure 5).

#### 3.4.4. Weight

**Figure 6:**
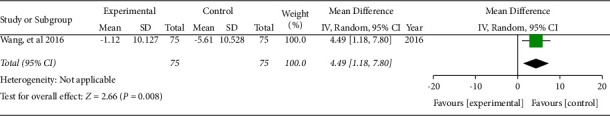
Forest plot of weight changes.

One literature included the weight changes. The combined effect was WMD = 4.49, 95% CI (1.18, 7.80), *P* < 0.05. The data were statistically significant (see Figure 6).

### 3.5. Numbers of Immune Cells

#### 3.5.1. CD3^+^

**Figure 7:**
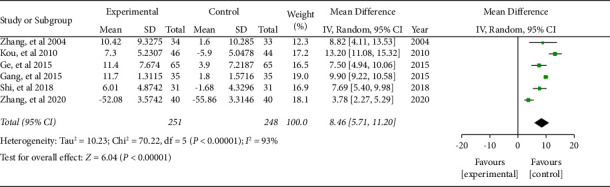
Forest plot of CD3^+^ cells.

Six literature included the numbers of CD3^+^ cells. The combined effect was WMD = 8.46, 95% CI (5.71, 11.20), *P* < 0.05. The data were statistically significant (see Figure 7).

#### 3.5.2. CD4^+^

**Figure 8:**
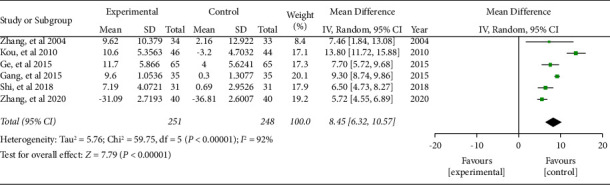
Forest plot of CD4^+^ cells.

Six literature included the numbers of CD4^+^ cells. The combined effect was WMD = 8.45, 95% CI (6.32, 10.57), *P* < 0.05. The data were statistically significant (see Figure 8).

#### 3.5.3. CD8^+^

**Figure 9:**
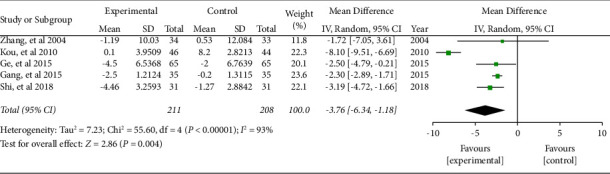
Forest plot of CD8^+^ cells.

Five literature included the numbers of CD8^+^ Cells. The combined effect was WMD = −3.76, 95% CI (−6.34, −1.18), *P* < 0.05. The data were statistically significant (see Figure 9).

#### 3.5.4. CD4^+^/CD8^+^

**Figure 10:**
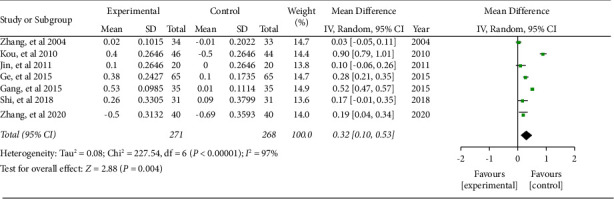
Forest plot of the ratio of CD4^+^/CD8^+^.

Seven literature included the ratio of CD4^+^/CD8^+^. The combined effect was WMD = 0.32, 95% CI (0.10, 0.53), *P* < 0.05. The data were statistically significant (see Figure 10).

#### 3.5.5. MDSC

**Figure 11:**
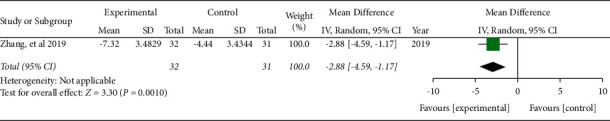
Forest plot of the numbers of myeloid-derived suppressor cells.

One literature included the numbers of myeloid-derived suppressor cells. The combined effect was WMD = −2.88, 95% CI (−4.59, −1.17), *P* < 0.05. The data were statistically significant (see Figure 11).

#### 3.5.6. NK

**Figure 12:**
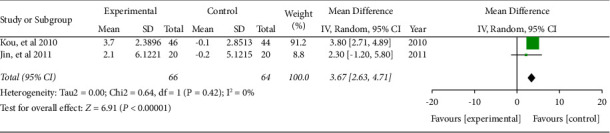
Forest plot of the numbers of natural killer cells.

Two literature included the numbers of natural killer cells. The combined effect was WMD = 3.67, 95% CI (2.63, 4.71), *P* < 0.05. The data were statistically significant (see Figure 12).

#### 3.5.7. Treg

**Figure 13:**
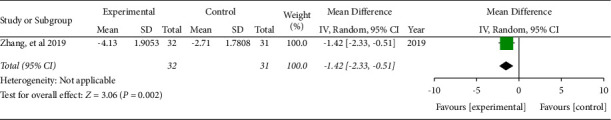
Forest plot of the numbers of Treg cells.

One literature included the numbers of Treg cells. The combined effect was WMD = −1.42, 95% CI (−2.33, −0.51), *P* < 0.05. The data were statistically significant (see Figure 13).

### 3.6. Levels of Cytokines and Secretions in Serum

#### 3.6.1. CEA

**Figure 14:**
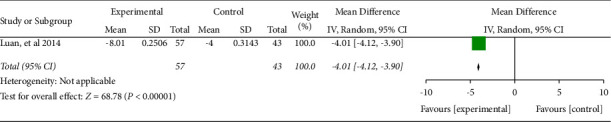
Forest plot of the level of CEA.

One literature included the level of CEA. The combined effect was WMD = −4.01, 95% CI (−4.12, −3.90), *P* < 0.05. The data were statistically significant (see Figure 14).

#### 3.6.2. NSE

**Figure 15:**
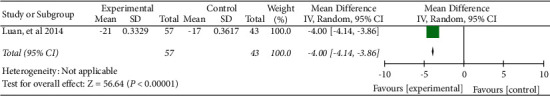
Forest plot of the level of NSE.

One literature included the level of NSE. The combined effect was WMD = −4.00, 95% CI (−4.14, −3.86), *P* < 0.05. The data were statistically significant (see Figure 15).

#### 3.6.3. IL-2

**Figure 16:**
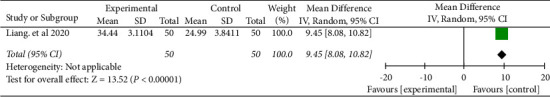
Forest plot of the level of IL-2.

One literature included the level of IL-2. The combined effect was WMD = 9.45, 95% CI (8.08, 10.82), *P* < 0.05. The data were statistically significant (see Figure 16).

#### 3.6.4. IL-4

**Figure 17:**
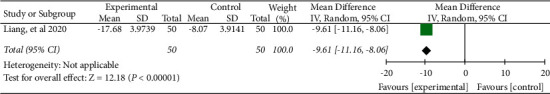
Forest plot of the level of IL-4.

One literature included the level of IL-4. The combined effect was WMD = −9.61, 95% CI (−11.16, −8.06), *P* < 0.05. The data were statistically significant (see Figure 17).

#### 3.6.5. IL-5

**Figure 18:**
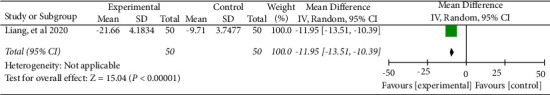
Forest plot of the level of IL-5.

One literature included the level of IL-5. The combined effect was WMD = −11.95, 95% CI (−13.51, −10.39), *P* < 0.05. The data were statistically significant (see Figure 18).

#### 3.6.6. IL-6

**Figure 19:**
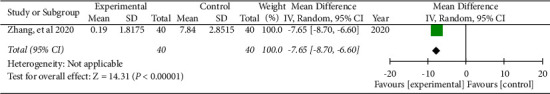
Forest plot of the level of IL-6.

One literature included the level of IL-6. The combined effect was WMD = −7.65, 95% CI (−8.70, −6.60), *P* < 0.05. The data were statistically significant (see Figure 19).

#### 3.6.7. IL-2/IL-5

**Figure 20:**
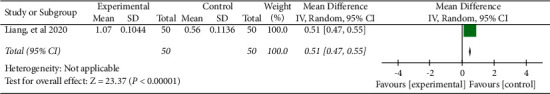
Forest plot of the ratio of IL-2/IL-5.

One literature included the ratio of IL-2/IL-5. The combined effect was WMD = 0.51, 95% CI (−0.47, 0.55), 95%, *P* < 0.05. The data were statistically significant (see Figure 20).

#### 3.6.8. IFN-*γ*

**Figure 21:**
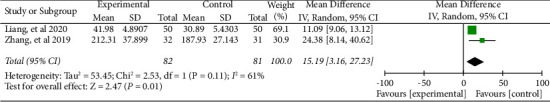
Forest plot of the level of IFN-*γ*.

Two literature included the level of IFN-*γ*. The combined effect was WMD = 15.19, 95% CI (3.16, 27.23), *P* < 0.05. The data were statistically significant (see Figure 21).

#### 3.6.9. IFN-*γ*/IL-4

**Figure 22:**
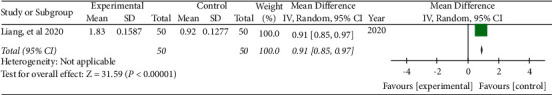
Forest plot of the ratio of IFN-*γ*/IL-4.

One literature included the ratio of IFN-*γ*/IL-4. The combined effect was WMD = 0.91, 95% CI (0.85, 0.97), *P* < 0.05. The data were statistically significant (see Figure 22).

#### 3.6.10. VEGF

**Figure 23:**
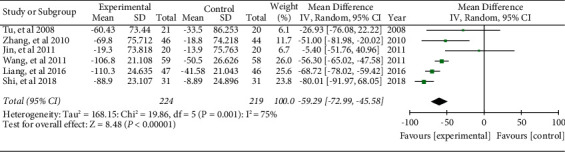
Forest plot of the level of VEGF.

Six literature included the level of VEGF. The combined effect was WMD = −59.29, 95% CI (−72.99, −45.58), *P* < 0.05. The data were statistically significant (see Figure 23).

#### 3.6.11. TGF-*α*

**Figure 24:**
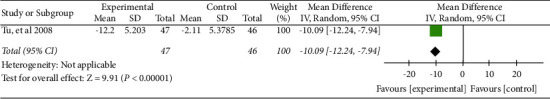
Forest plot of the level of TGF-*α*.

One literature included the level of TGF-*α*. The combined effect was WMD = −10.09, 95% CI (−12.24, −7.94), *P* < 0.05. The data were statistically significant (see Figure 24).

#### 3.6.12. TGF-*β*

**Figure 25:**
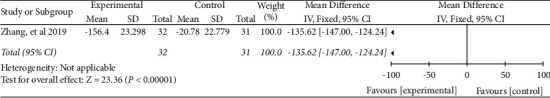
Forest plot of the level of TGF-*β*.

One literature included the level of TGF-*β*. The combined effect was WMD = −135.62, 95% CI (−147.00, −124.24), *P* < 0.05. The data were statistically significant (see Figure 25).

#### 3.6.13. TGF-*β*1

**Figure 26:**
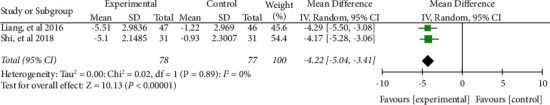
Forest plot of the level of TGF-*β*1.

Two literature included the level of TGF-*β*1. The combined effect was WMD = −4.22, 95% CI (−5.04, −3.41), *P* < 0.05. The data were statistically significant (see Figure 26).

#### 3.6.14. Arginase

**Figure 27:**
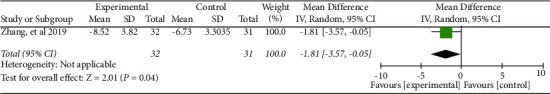
Forest plot of the level of arginase.

One literature included the level of arginase. The combined effect was WMD = −1.81, 95% CI (−3.57, −0.05), *P* < 0.05. The data were statistically significant (see Figure 27).

#### 3.6.15. IgG

**Figure 28:**
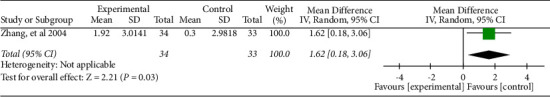
Forest plot of the level of IgG.

One literature included the level of IgG. The combined effect was WMD = 1.62, 95% CI (0.18, 3.06), *P* < 0.05. The data were statistically significant (see Figure 28).

#### 3.6.16. IgM

**Figure 29:**
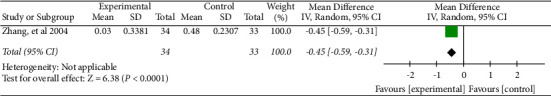
Forest plot of the level of IgM.

One literature included the level of IgM. The combined effect was WMD = −0.45, 95% CI (−0.59, −0.31), *P* < 0.05. The data were statistically significant (see Figure 29).

## 4. Discussion

### 4.1. Summary of Main Findings

Ginseng, as the representative of traditional Chinese medicine for tonifying qi, is a complementary and alternative medicine approved by the National Institutes of Health of the United States. The anticancer function of ginseng has been increasingly recognized in clinical practice, and the underlying mechanism could be related to the regulation of body immunity. Nevertheless, the evidence supporting its efficacy and safety is still insufficient. This study includes 1480 cases in 17 RCT studies. All the studies use ginseng in combination with chemotherapy versus chemotherapy alone in NSCLC patients. Most of the studies have a low risk of bias, while all of them do not mention performance bias and detection bias. The results of the integration of clinical outcomes showed that the treatment of ginseng (or combination of ginseng with chemotherapy) can improve the quality of life of patients with NSCLC and promote an antitumor response. In addition, a reduction of the inflammatory level and an increase of antitumor indicators in serum were also reported. The meta-analysis result shows the following: Karnofsky score: WMD = 16, 95% CI (9.52, 22.47); quality-of-life score: WMD = 8.55, 95%CI (6.08, 11.03); lesion diameter: WMD = −0.45, 95% CI (−0.75, −0.15); weight: WMD = 4.49, 95% CI (1.18, 7.80); CD3^+^: WMD = 8.46, 95% CI (5.71, 11.20); CD4^+^: WMD = 8.45, 95% CI (6.32, 10.57); CD8^+^: WMD = −3.76, 95% CI (−6.34, −1.18); CD4^+^/CD8^+^: WMD = 0.32, 95% CI (0.10, 0.53); MDSC: WMD = −2.88, 95% CI (−4.59, −1.17); NK: WMD = 3.67, 95% CI (2.63, 4.71); Treg: WMD = −1.42, 95% CI (−2.33, −0.51); CEA: WMD = −4.01, 95% CI (−4.12, −3.90); NSE: WMD = −4.00, 95% CI (−4.14, −3.86); IL-2: WMD = 9.45, 95% CI (8.08, 10.82); IL-4: WMD = −9.61, 95% CI (−11.16, −8.06); IL-5: WMD = −11.95, 95% CI (−13.51, −10.39); IL-6: WMD = −7.65, 95% CI (−8.70, −6.60); IL-2/IL-5: WMD = 0.51, 95% CI (0.47, 0.55); IFN-*γ*: WMD15.19, 95% CI (3.16, 27.23); IFN-*γ*/IL-4: WMD = 0.91, 95% CI (0.85, 0.97); VEGF: WMD = −59.29, 95% CI (−72.99, −45.58); TGF-*α*: WMD = −10.09, 95% CI (−12.24, −7.94); TGF-*β*: WMD = −135.62, 95% CI (−147.00, −124.24); TGF-*β*1: WMD = −4.22, 95% CI (−5.04, −3.41); arginase: WMD = −1.81, 95% CI (−3.57, −0.05); IgG: WMD = 1.62, 95% CI (0.18, 3.06); IgM: WMD = −0.45, 95% CI (−0.59, −0.31). All results are statistically significant. No adverse events were reported in the included articles.

### 4.2. Applicability of the Current Evidence

Lesion diameter is the most favorable evidence to explain the effect of drug treatment. According to the results, ginseng can remarkably reduce the lesion volume of NSCLC patients, suggesting the feasibility of ginseng as an adjuvant therapy for cancer. The Karnofsky score is a kind of standard to describe the body's function and tolerance to the treatment. A higher score indicates better physical function and higher tolerance. Among the results of our systematic review and meta-analysis, ginseng and its active components significantly improved the Karnofsky score. Additionally, the quality-of-life score and weight, which represent the quality of life of patients, were increased by ginseng. These data revealed the advantages of ginseng compared with chemotherapy drugs.

T cells and NK cells are the main killer immune cells for the body to resist virus infection and tumorigenesis. In a large number of experimental studies, the antitumor immune response of T cells and NK cells is emphasized [47–50]. Myeloid-derived suppressor cells and Treg cells are often associated with immunosuppression. For example, myeloid-derived suppressor cells can secrete arginase to inhibit the antitumor activity of immune cells and secrete TGF-*β* to promote tumor growth [51, 52], as a result, it promotes the development of tumors and leads to the deterioration of patients' tumors. In addition, studies have shown that VEGF, TGF-*α*, and TGF-*β*1 play an important role in promoting tumor angiogenesis and tumor growth [53–55]. Although the use of chemotherapeutic drugs has a significant effect on inhibiting tumor growth, it will cause a sharp decrease in the patient's immune cells and affect the patient's immune function. Ginseng has the ability to regulate immunity. Through the above analysis, we find that the combined use of ginseng and chemotherapy increases the number of CD3^+^, CD4^+^T cells, and NK cells in NSCLC patients. It also increases the ratio of CD4^+^/CD8^+^ T cells and increases serum immunoglobulin IgG, reduces the number of myeloid-derived inhibitory cells and regulatory T cells, and decreases serum arginase, TGF-*β*, VEGF, TGF-*α*, and TGF-*β*1 levels. The increase of CEA and NSE in serum is usually used for the clinical diagnosis of non-small cell lung cancer, and the increase in CEA level is often closely related to the metastasis and infiltration of non-small cell lung cancer [56].

In our research, we find that the levels of CEA and NSE in the serum were significantly reduced after using ginseng and its active components. Th1 and Th2, the two types of CD4^+^ T cells, have diametrically opposite roles in tumors. The Th1 phenotype can secrete IFN-*γ*, IL-2, and other factors to fight tumors, but IL-4 and IL-5 secreted by the Th2 phenotype have tumor-promoting effects. Therefore, the occurrence of tumors often leads to Th1/Th2 immune imbalance [57–59]. Our analysis shows that after adjuvant chemotherapy with ginseng and its active components, patients' IFN-*γ* and IL-2 are both increasing while IL-4 and IL-5 are decreasing. Using IFN-*γ*/IL-4 and IL2/IL-5 as indicators of Th1/Th2 balance, it is found that the treatment of ginseng and its active components can help restore the Th1/Th2 phenotype. Most literature shows that inflammation tends to promote the progression of cancer [60, 61]. One study has found that IL-6, as a proinflammatory factor, can promote cancer metastasis [62]. We also found that the level of IL-6 decreased after using ginseng and its active components, which indicates that ginseng and its active components are helpful for antitumor treatment. It was recently reported that the underlying mechanism may involve the inhibition of STAT3/PD-L1 and the activation of miR193a-5p [13]. Therefore, we consider that ginseng and its active components are helpful for NSCLC patients' conditions, immune cells, cytokines, and secretions in serum.

### 4.3. Limitations of This Review

This study has several limitations. First, the quality of the included RCTs is generally common according to Cochrane's risks of bias tool. Most studies did not mention the performance bias and detection bias. Second, the types of chemotherapy combined with ginseng are different. Due to the lack of relevant literature, subgroup analysis was not carried out. Third, our analysis was based on 17 RCTs, and most of them had a relatively small sample size (*n* < 100). In addition, ginseng is a traditional Chinese medicine, which is widely used in China. All 17 included trials were written in Chinese, and none of the included trials mentioned adverse events. Last but not least, the follow-up periods of most studies are too short to observe the survival rate. We cannot assess the long-term function of ginseng and its active components. Therefore, well-conducted RCTs are urgently needed to evaluate the efficacy and hemorheological indexes of ginseng and its active components on non-small cell lung cancer.

## 5. Conclusion

It is a reasonable choice to use ginseng and its active components as adjuvant therapy for NSCLC. Ginseng is helpful for NSCLC patients' conditions, immune cells, cytokines, and secretions in the serum. There is still a need for increasing RCTs about changes in patients' conditions, numbers of immune cells, and levels of cytokines and secretions in serum to address whether ginseng and its active components are effective on NSCLC.

## Figures and Tables

**Table 1:** Characteristics of the study.

Author (Year)	Experience group average age	Experience group number	Control group average age	Control group number	Experience group method	Control group method	Research designs
Zhang et al. (2004) [30]		34		33	Compound ginseng polysaccharide, chemotherapy, and radiotherapy	Chemotherapy and radiotherapy	RCT
Sun et al. (2006) [31]	59.54	54	57.44	61	Shenyi capsule and NP chemotherapy	NP chemotherapy	RCT
Tu (2008) [32]		20		21	Ginsenoside Rg3, paclitaxel, and cisplatin	Paclitaxel and cisplatin	RCT
Kou et al. (2010) [33]		46		44	Ginsenoside Rg3, gemcitabine, and cisplatin	Gemcitabine and cisplatin	RCT
Zhang et al. (2010) [34]		46		44	Ginsenoside Rg3, gemcitabine, and cisplatin	Gemcitabine and cisplatin	RCT
Wang et al. (2011) [35]		59		58	Shenyi capsule, gemcitabine, and cisplatin or Shenyi capsule, vinorelbine, and cisplatin	Gemcitabine and cisplatin or vinorelbine and cisplatin	RCT
Jin et al. (2011) [36]		20		20	Shenyi capsule, gemcitabine, and cisplatin	Gemcitabine and cisplatin	RCT
Luan (2014) [37]		57		43	Shenyi capsule, gemcitabine, and cisplatin	Gemcitabine and cisplatin	RCT
Ge et al. (2015) [38]	60.8	67	59.4	75	Ginseng polysaccharide, gemcitabine, and cisplatin	Gemcitabine and cisplatin	RCT
Gang (2015) [39]	63.33	35	63.35	35	Ginseng and sodium cantharidinate vitamin B6	Sodium cantharidinate vitamin B6	RCT
Wang (2016) [40]	67.49	75	65.67	75	Ginseng polysaccharide, pemetrexed, and cisplatin or ginseng polysaccharide, gemcitabine, and cisplatin	Pemetrexed and cisplatin or gemcitabine and cisplatin	RCT
Liang and Han (2016) [41]	67.47	47	66.32	46	Shenyi capsule, gemcitabine, and cisplatin	Gemcitabine and cisplatin	RCT
Shi (2018) [42]	69.67	31	68.34	31	Shenyi capsule, paclitaxel, and carboplatin	Paclitaxel and carboplatin	RCT
Zhang et al. (2019) [43]	62.8	32	61.7	31	Ginseng polysaccharide, gemcitabine, and cisplatin or ginseng polysaccharide, docetaxel, and cisplatin	Gemcitabine and cisplatin or docetaxel and cisplatin	RCT
Jiang et al. (2019) [44]		30		30	Ginsenoside Rg3 and osimertinib	Osimertinib	RCT
Zhang et al. (2020) [45]	45.23	40	47.13	40	Shenyi capsule, gemcitabine, and cisplatin	Gemcitabine and cisplatin	RCT
Liang et al. (2020) [46]	64.1	50	62.5	50	Ginseng polysaccharide, gemcitabine, and cisplatin	Gemcitabine and cisplatin	RCT

## Data Availability

No primary data in this article.

## Abbreviations

NSCLC: Non-small cell lung cancer
RCTs: Randomized controlled trials
CD3^+^: CD3^+^ pan T cells
CD4^+^: CD4^+^ pan T cells
CD8^+^: CD8^+^ pan T cells
MDSC: Myeloid-derived suppressor cells
NK: Nature killer cells
Treg: Regulatory T cells
CEA: Carcinoembryonic antigen
NSE: Neuron-specific enolase
IL-2: Interleukin-2
IL-4: Interleukin-4
IL-5: Interleukin-5
IL-6: Interleukin-6
IFN-*γ*: Interferon-*γ*
VEGF: Vascular endothelial growth factor
TGF-*α*: Transforming growth factor-*α*
TGF-*β*: Transforming growth factor-*β*
TGF-*β*1: Transforming growth factor-*β*1
IgG: Immunoglobulin G
IgM: Immunoglobulin M.

## Supplementary Materials

Supplementary Materials PRISMA 2009 Checklist.
